# Neuromodulation and Cognitive Rehabilitation: Addressing the Methodological Issue of Circadian Rhythms

**DOI:** 10.3389/fpsyt.2014.00150

**Published:** 2014-10-27

**Authors:** Massimiliano Gobbo, Luca Falciati

**Affiliations:** ^1^Department of Clinical and Experimental Sciences, University of Brescia, Brescia, Italy; ^2^Laboratory of Neuromuscular Rehabilitation, Teresa Camplani Foundation, Brescia, Italy

**Keywords:** circadian-dependent plasticity, neural connectivity, cognitive performance, neuropsychological assessment, personalized medicine

Most organisms, including humans, exhibit daily rhythms in their biological activities, physiological functions, and homeostatic mechanisms such as cell regeneration, hormone production, cardiac output, blood pressure, blood flow distribution, and body temperature. The physiological system responsible for these rhythms is known as the circadian system.

Circadian changes have increasingly become an interesting focus of research, concerning also neurobehavioral functioning of healthy subjects. The impact of factors such as the sleep–wakefulness cycle and biological time-of-day on measures of subjective alertness has been extensively studied (1, 2). Moreover, there is compelling evidence of circadian dependency also for cognitive functions such as attention, memory, and learning (3, 4).

Recently, it has been consolidated in different experimental models, including mammalian brain, that the circadian clock has a role in regulating structural synaptic plasticity, opening the new relevant concern of circadian-dependent neural plasticity (5, 6). Interestingly, it has been suggested that changes in the electrical properties of the cell membrane (intrinsic plasticity) and in the release of neuromodulatory molecules due to the internal clock can reconfigure circuit dynamics leading individual neurons to switch among different functional networks throughout the day (5). Daily rhythmicity in neural activity has been further elucidated by Blautzik et al. (7) who analyzed the daily course of connectivity patterns. The authors found different degrees of daily modulation across connectivity patterns, ranging from networks characterized by stable activity across the day and networks with highly rhythmic connectivity changes. Based on the reported findings, we can infer that the aforementioned oscillatory processes in connectivity strength and spatial extent would eventually determine highly individual fluctuations of effective connectivity over the course of the day.

Circadian rhythms show also to exert influence on the excitability of the cerebral cortex, as found by Lang et al. (8). In this study, the excitability of the primary motor cortex (M1) of healthy subjects was evaluated by transcranial magnetic stimulation (TMS) at different times of the day. Data unveiled that both the intracortical and the corticospinal excitability of M1 exhibited a progressive decrease during the course of the day.

In the last years, it has emerged that the effectiveness and reproducibility of several techniques able to induce neuroplastic changes in humans, such as paired associative stimulation (PAS), are influenced by time-of-day of the intervention (9) and subjected to circadian modulation. As demonstrated by Sale et al. (10), who tested 25 subjects twice, at 8:00 a.m. and 8:00 p.m., on separate days, PAS effectiveness is enhanced in the evening, when endogenous cortisol is low; conversely, effects of PAS in the evening are blocked by a single oral dose of hydrocortisone.

Additionally, in a large study of humans aged 50–70 years, high salivary levels of cortisol appeared to be related with poor performances on a wide range of cognitive domains, including language, verbal learning, processing speed, memory, and eye–hand coordination (11).

Overall, the circadian modulation of several neural properties and structures, at both the microscopic and functional levels, may deeply affect cognitive behavior, responsiveness, and performance within the day. Despite its potential impact, time-of-day is rarely contemplated when brain responses and cognitive functions are studied. As a matter of fact, in order to minimize possible biases related to circadian effects, some neurophysiological studies are conducted with evaluations and/or interventions performed at the same time of the day. Still, these experimental designs do not contemplate another relevant factor, which may strongly affect the reliability of the collected data that is the interindividual variability of the biological clock. This physiological variability of circadian rhythms between subjects has led to the notion of individual chronotypes (12, 13). The chronotype influences the organization of physiological functions, behaviors, and cognitive performances throughout the day (1, 14, 15). Given the differences in circadian rhythmicity between chronotypes, specific individual variations in task performance are likely to occur as a function of time-of-day. In other words, this implies that the scheduled task may not be necessarily synchronized to the most optimal moment in the day for each tested participant (16, 17).

The regulation of the endogenous dynamics that characterizes a chronotype is dictated by many factors. The thorough understanding of these mechanisms is critical to gain a comprehensive view on their functional implications and, further, may be considerably useful when addressing the experimental limitations due to diurnal rhythmicity. Among them, cortisol is a main neuromodulator that mediates circadian processes. The normal diurnal pattern of cortisol secretion has been fairly well characterized: its plasma concentration rises quickly after awaking in the morning (cortisol awakening response) and starts declining about 60 min after waking (18) with a progressive decrement during the afternoon and the evening to a nadir ~14 h after awaking (19). The diurnal changes of cortisol, which remarkably are correlated with daily variations of BDNF (20) and cerebral blood flow (18, 21, 22), support the existence of a circadian trend of cognitive performance (4). With specific regards to daily fluctuations in hemodynamic parameters, Hodkinson et al. (18) have recently observed that changes in regional cerebral blood flow within the anterior cingulate cortex were closely correlated with functional connectivity and recommend to put particular attention to possible strong circadian bias especially in the morning. Another factor to consider is the variation in regional brain glucose metabolism during the day (23). High blood glucose level could result in a stronger activation of the hypothalamic–pituitary–adrenal axis, thus mediating the cortisol response and subsequently affecting cognitive performances, such as improved ability to retain new information and to recall old memories, and vigilance (24). For its possible impact in the context of neuropsychological assessment, it should be taken into account that Micha et al. (24) found a significant decay of glucose effects on cognitive function approximately 2.5 h after food intake.

Up to this point, we have referred to healthy young and adults. A different condition pertains to elderly people since dysregulation of the circadian clock represents a natural process of aging (25). Alterations in the endogenous circadian system become even more pronounced when considering individuals presenting neurological and/or psychiatric disorders (4). In this regard, literature data outline that serious disruption in sleep–wake rhythmicity and diurnal endogenous dynamics is typical of Alzheimer’s disease, Parkinson’s disease, Huntington’s disease, major depression, bipolar disorders, stroke, and traumatic brain injury (4, 26, 27).

According to the previously discussed evidence, we can argue that when dealing with cognitive rehabilitation it is of paramount importance to reckon with pronounced abnormal daily fluctuations of physiological functions and cognitive performance. In other words, these concerns have potential implications for research and clinical practice in terms of contrasting results/outcomes stemming from differences in patient’s chronotypes and inappropriate timing of assessment or treatment delivery.

To overcome this methodological issue some solutions are suggested. Salivary cortisol monitoring probably represents the most precise and reliable marker for the internal pacemaker, which could allow for a convenient characterization of the individual chronotype. Salivary cortisol sampling has become increasingly common over the past few years since collecting saliva represents an easy modality, which, moreover, can be repeated at frequent intervals (18). On the other hand, this approach might be less affordable for the clinical context and with limited cost-effectiveness. Other methods could be suggested as suitable solutions to identify individual circadian patterns. Mental chronometry offers a lot of paradigms, which are widely applied to investigate the cognitive functioning. Speculatively, reaction times may represent a reliable index to define the circadian profile of each patient when measured for monitoring diurnal oscillations of cognitive abilities. Alternatively, body temperature measurements may also be used to track the endogenous rhythm for their feasibility especially in clinical settings.

This patient-tailored approach based on personal chronobiology is recommended in the context of modern advanced cognitive rehabilitation, in particular when neuromodulation techniques are provided to harness at best neural plasticity.

In conclusion, converging evidence from neurophysiological and neuropsychological literature indicates that cognitive ability varies as a function of the circadian processes, which may lead to discrepancy between the critical time windows for individual best cognitive performance and the time of diagnostic assessments or rehabilitative interventions scheduled during the day. Cognitive performance may thus be enhanced or impaired depending on when it is measured. This methodological issue should be carefully addressed when designing research studies in order to collect reliable experimental data and limit misinterpretation due to inherent rhythmicity and individual chronotype. A careful control for the time-of-day effects is recommended also to attain consistent and possibly better treatment outcomes in clinical settings, thus pursuing and fostering the emerging and highly desirable model of personalized medicine.

## Conflict of Interest Statement

The authors declare that the research was conducted in the absence of any commercial or financial relationships that could be construed as a potential conflict of interest.
